# An Overview of Nanocarrier-Based Adjuvants for Vaccine Delivery

**DOI:** 10.3390/pharmaceutics13040455

**Published:** 2021-03-27

**Authors:** Kailash C. Petkar, Suyash M. Patil, Sandip S. Chavhan, Kan Kaneko, Krutika K. Sawant, Nitesh K. Kunda, Imran Y. Saleem

**Affiliations:** 1 Department of Scientific and Industrial Research, Ministry of Science & Technology, Government of India, New Delhi 110016, India; 2 Department of Pharmaceutical Sciences, College of Pharmacy and Health Sciences, St. John’s University, Jamaica, NY 11439, USA; suyash.patil19@my.stjohns.edu; 3 Department of Pharmaceutics, Faculty of Pharmacy, The Maharaja Sayajirao University of Baroda, Kalabhavan, Vadodara 390001, India; yuvraj1021@gmail.com (S.S.C.); krutikasawant@yahoo.co.in (K.K.S.); 4 School of Pharmacy and Biomolecular Sciences, Liverpool John Moores University, Liverpool L3 3AF, UK; K.Kaneko@ljmu.ac.uk

**Keywords:** nanotechnology, nanocarriers, adjuvants, vaccines, vaccine delivery systems, viral vectors, non-viral vectors

## Abstract

The development of vaccines is one of the most significant medical accomplishments which has helped to eradicate a large number of diseases. It has undergone an evolutionary process from live attenuated pathogen vaccine to killed whole organisms or inactivated toxins (toxoids), each of them having its own advantages and disadvantages. The crucial parameters in vaccination are the generation of memory response and protection against infection, while an important aspect is the effective delivery of antigen in an intelligent manner to evoke a robust immune response. In this regard, nanotechnology is greatly contributing to developing efficient vaccine adjuvants and delivery systems. These can protect the encapsulated antigen from the host’s in-vivo environment and releasing it in a sustained manner to induce a long-lasting immunostimulatory effect. In view of this, the present review article summarizes nanoscale-based adjuvants and delivery vehicles such as viral vectors, virus-like particles and virosomes; non-viral vectors namely nanoemulsions, lipid nanocarriers, biodegradable and non-degradable nanoparticles, calcium phosphate nanoparticles, colloidally stable nanoparticles, proteosomes; and pattern recognition receptors covering c-type lectin receptors and toll-like receptors.

## 1. Introduction and Historical Background

Nanotechnology used for the diagnosis, monitoring, control, prevention and treatment of disease is referred to as “nanomedicine” [1]. Nanomedicine is a vast area that includes the use of nanoscale materials for a wide spectrum of applications from sensing, laboratory diagnostics (e.g., quantum dots) [2] to silicon microchips for drug release, micro-machined hollow needles (e.g., nanoscale microfabrication-based devices), etc. In recent years, an interesting application of nanotechnology in vaccine delivery has been exploited [3,4].

The development of vaccines is one of the most successful interventions that has helped to decimate many diseases. The Centre for Disease Control and Prevention (CDC) defines a vaccine as a product that stimulates a person’s immune system to produce immunity to a specific disease, protecting the person from that disease [5]. Vaccines stimulate the host’s immune system to recognize, destroy and remember the microbe and fight against the same microbial infection in the future. The development of vaccines has helped to completely eradicate certain diseases such as smallpox globally [6] and polio in many parts of the world (except Afghanistan and Pakistan where wild poliovirus transmission is reported) [7,8], whereas millions of death and disabilities due to diphtheria, measles, pertussis, poliomyelitis, tetanus, and tuberculosis have been prevented since the establishment of the Expanded Programme on Immunization (EPI) by WHO in 1974 [9]. Other diseases like influenza and infections due to hepatitis B virus (HBV) and pneumococci are partially controlled by vaccines [9]. Vaccine development is an extraordinary journey, which started from live-attenuated pathogen vaccine to killed whole organisms or inactivated toxins (toxoids) and most recent r-DNA and subunit vaccines, each of them possesses some benefits and shortcomings [10]. Since a live-attenuated vaccine introduces an actual pathogen to the host, it represents an exact simulation of the immune response leading to life-long immunity with just one or two doses. However, such vaccines may not be suitable for administration to immunocompromised patients due to risks associated with them. The risk, in this case, is due to the fact that a person who receives live-attenuated vaccine may occasionally cause transmission of vaccine strain to others (particularly a family member or healthcare worker who provide care to immunocompromised patients) [11]. Furthermore, recombinant DNA vaccines, subunit vaccines and conjugate vaccines have become more attractive due to their safety (as unlike live-attenuated vaccines they cannot revert back to a virulent form) and feasibility to produce large quantities of antigen from recombinant methods (i.e., it is easy to scale up for mass production) [12]. However, these vaccines generate a weak immune response due to the use of only a specific part of the whole organism, while the basic requirement of a potential vaccine is that it should produce a sufficient immunogenic response to provide effective protection to a host with minimum side effects. In this context, adjuvants and delivery systems are often necessary to deliver antigen in a proper manner and to provoke a robust immune response.

Adjuvants are substances that, when used in combination with vaccine antigens, induce a stronger and more efficacious response to the vaccine as compared to that induced by the vaccine alone [13]. The addition of adjuvants to vaccines has been used to (i) enhance the immunogenicity of antigens; (ii) reduce the amount of antigen or the number of immunizations needed for protective immunity; (iii) improve the efficacy of vaccines in newborns, the elderly or immuno-compromised persons [14]. Broadly, adjuvants have been grouped into two classes, “immunostimulatory adjuvant” and “vaccine delivery systems”. Immunostimulatory adjuvants (immunopotentiators) are derived from pathogen representing pathogen-associated molecular patterns (PAMP) (e.g., lipopolysaccharides (LPS), monophosphoryl lipid (MPL), cytosine phosphoguanine deoxynucleoties (CpG DNA)) and initiate innate immunity directly (through cytokines) or through pattern recognition receptors (PRRs), whereas delivery systems concentrate and display antigens, target vaccine antigens to antigen-presenting cells (APCs) and help co-localize antigens and immunopotentiators. For many years, Alum (aluminum hydroxide or aluminum phosphate gels) has been used as an adjuvant in several licensed vaccines, which eliminates many cytotoxic properties of Incomplete Freund’s adjuvant (IFA) [9,15]. However, it suffers from drawbacks like instability to freezing and drying, inconsistent and weak humoral immunity and certain safety concerns which makes it somewhat inefficient immunostimulatory [16]. In this regard, delivery systems are greatly contributing to the development of efficient vaccine adjuvants which acts by facilitating the antigen uptake by APCs or by elevating the influx of APCs. One advantage of delivering vaccines in particulate formulations is the ability to protect the antigens from proteolytic breakdown thereby enhancing vaccine uptake by the cells [17]. Immunopotentiators, e.g., PRRs may be employed along with the delivery system as they are capable of recognizing molecules frequently present in pathogens and help generate a robust immune response. Thus, the delivery system protects the encapsulated antigen from the host’s in-vivo environment and releases it in a sustained manner to induce a long-lasting target specific immune response, while immunostimulatory adjuvants (PRRs) provides a stimulatory amount of linked PRR ligand and enhanced uptake of linked antigen for its effective and preferential presentation to APC. This combined approach certainly provides prolong antigen delivery with increased immunogenicity.

**Figure 1:**
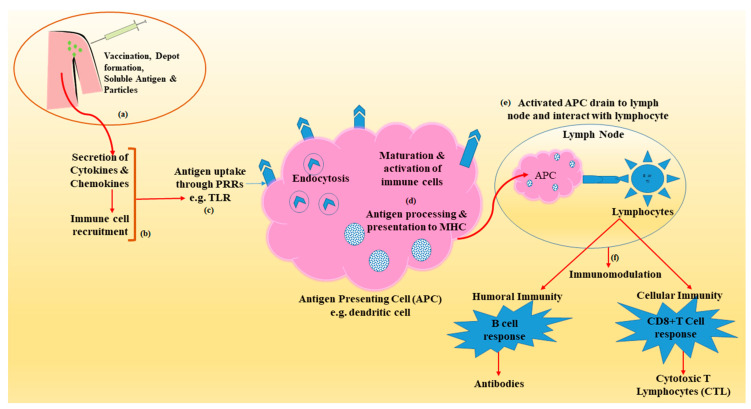
Representative mechanism of action of adjuvants. (**a**) After vaccination, depot formation (in some cases), release of particles and soluble antigen; (**b**) Secretion of cytokines and chemokines, which are involved in the recruitment of various immune cells at the injection site thereby formation of immunocompetent environment; (**c**) Antigen uptake through various pattern recognition receptors (PRRs), e.g., Toll-like receptor (TLR) which are expressed on surface as well as intracellularly. These PRRs are recognized and activated by the antigens and adjuvants; (**d**) Antigen uptake by antigen-presenting cells (APCs) leads to maturation and activation of immune cells and further processing for presentation to major histocompatibility complex (MHC) by upregulating the expression of MHC; (**e**) Activated APCs then migrate to draining lymph node to interact with lymphocyte; (***f***) this leads to immunomodulation by triggering humoral and cellular immunity [19,20].

Advanced immunological research has revealed several mechanisms by which vaccine adjuvants and delivery vehicles elicit humoral and cellular immunity (Figure 1) [9]. This may include a combination of one or more of the several mechanisms such as depot formation, upregulation of cytokine and chemokine, immune cell recruitment at the site of injection, enhanced antigen uptake and presentation to the APCs and surface and intracellular expression of PRRs, activation of inflammasomes, activation and maturation of APCs (upregulation of expression of major histocompatibility complex (MHC) and co-stimulatory molecules), migration of matured APC to draining lymph node and immunomodulation through induction of B cell (antibodies or immunoglobulins) and T cell (cytotoxic T lymphocyte-CTL) (Table 1) [18,19].

The ability of an adjuvant to qualitatively trigger the immune response is of prime importance, considering the need for vaccines against cancer, chronic infections such as HIV, Hepatitis C Virus, Tuberculosis, herpes simplex virus (HSV) and severe acute respiratory syndrome coronavirus 2 (SARS-CoV-2). An extended comprehension of the immunobiology of Toll-like receptors (TLRs) and other PRRs, immunoregulatory cells, and the importance of specific T helper (Th) cell responses in resolving specific diseases, provides a necessary framework for the continued development and optimization of vaccines. This review summarizes various nanosized vaccines (viral), adjuvants and non-viral vectors (delivery systems) with their recent clinical applications. 

## 2. Nanotechnology-Based Vaccines, Vaccine Adjuvants and Delivery Systems

The introduction of nanotechnology for the development of nanocarriers (adjuvants and delivery systems) in delivering vaccines is one of the most exciting and challenging applications in medicine. Nanotechnology in this context refers to the nanocarriers on the nanoscale (i.e., 1–100 nm). Nanocarriers including viral and non-viral vectors have been studied as potential tools to deliver vaccines as they are expected to elicit a broad range of immune responses including cell-mediated immunity (CMI). CMI is an immune response that involves the activation of macrophages and natural killer (NK) cells, the production of antigen-specific cytotoxic T-lymphocytes (CTL), and the release of various cytokines in response to an antigen [40]. Activation of the macrophages and NK cells helps to destroy intracellular pathogens, the release of CTL destroys cells that display foreign epitopes on their surface and various cytokines are responsible for the regulation of immune response [40]. Thus, CMI helps in one way or another to destroy the infected cells or bacteria/virus. In this article, viral vector-based vaccines, adjuvants and non-viral particles and delivery systems are reviewed due to their widespread application in vaccine delivery. Nanocarriers offer many advantages, most importantly their capacity to interact with biological barriers and transport the bioactive molecule without altering its antigenicity. Moreover, they—(i) present a large amount of antigen to the immune system; (ii) control/sustain release of the antigen over a prolonged period of time; (iii) limit the adverse effects associated with adjuvants by restricting their distribution; (iv) provide stability to antigen by protecting it from degradation, and (v) provide immunomodulation and/or immunopotentiation, etc. confirming their utility in vaccinology [41,42]. Following sections deals with the comprehensive overview of viral vectored vaccines, adjuvants and delivery systems.

### 2.1. Viral Vectored Vaccines

The concept of viral vectors was introduced by Jackson et al., in 1972 to create recombinant DNA from the CV40 virus using genetic engineering [26]. Thereafter, several viral vectors have been studied and developed for the benefit of mankind [23,24,26,43]. A viral vector is a naturally evolved vehicle (from virus) and a promising tool for the purpose of introducing genetic material into the host cell for gene delivery and vaccines to initiate immune responses [26]. Several viral vectors have been discovered to date. Every viral vector has distinct advantages such as long term gene expression (Adenovirus, retrovirus, lentivirus), high titer production (vaccinia and adenovirus), infects non-dividing and dividing cells (lentivirus), high immunogenicity (lentivirus, vaccinia, Sendai virus, etc.), induction of unique CTL response (adeno), etc., while disadvantages include low titer production (adeno-associated adenovirus), generation of replication-competent virus (retrovirus, lentivirus), the potential for tumorigenesis (retrovirus, lentivirus), infects dividing cells only (retrovirus) [44]. In addition, viral vectors present some common advantages such as stability of the genome, ease of production, cost-effectiveness, cell specificity over live/attenuated vaccines, ability to deliver multiple immunogenic with efficient expression and strong immune response [9,25]. It is well known that the presentation of proteins to the immune system using viral vectors is similar to those occurring in a natural infection cycle and therefore provides potent induction of cellular and humoral immune responses [19]. Viral vectors have been playing a vital role in the development of vaccines due to their unique properties such as encapsulation and protection of sensitive compounds, ease in modification of exterior, their innate biocompatibility and naturally immunogenic etc. which led to their developments as actively targeted drug delivery systems [41,42]. Various types of viral vectors like retrovirus, lentivirus, adenovirus, adeno-associated virus, herpesvirus, poxvirus, vesicular stomatitis virus, alphavirus, measles virus, poliovirus, cytomegalovirus, Sendai virus and HBV have been employed in clinical/preclinical trials due to their ability to induce a robust immune response and enhanced cellular immunity [26,43]. However, they harbor risks such as toxicities, immunoresponses towards viral antigens or potential viral recombination, large or complex construction, which limit their clinical use [26]. Although the use of viral vectors is a promising system for developing effective and safe vaccines against many diseases, more studies are needed before the ideal vector is developed and licensed for human use. 

Viral vector such as adenovirus [27], modified vaccinia virus Ankara (MVA) [45], canary pox vector [46], pox virus [47], measles virus [44], herpes simplex virus (HSV) [48], alpha virus [49] have been pursued for vaccine delivery against various diseases. Among them, adenovirus and vaccinia virus are the most widely used vectors. They have provided an excellent delivery platform for many vaccines against influenza [50], tetanus [51], malaria [52], human immunodeficiency virus (HIV) [53] and COVID-19 [54] for different routes of administration. Literature reveals that the human adenoviral vectors (Ad26, Ad35, Ad5, etc.) induced enhanced memory CD8+ T cells and more polyfunctional CD8+ T cells and have been evaluated in clinical trials [55]. Vaccination of Ad26 priming vector with an MVA boost elicited a strong and durable antibody response to the Ebola virus antigen [56,57]. Human Ad serotype 5 (Ad5) vector has also been widely investigated as a gene delivery vector due to its ability to produce high titers [26]. In a study carried out by Zhu et al., a recombinant Ad5-based vaccine expressing the glycoprotein of Ebola Zaire Makona variant was evaluated in phase II clinical trials based on the promising results of previous preclinical and phase I clinical trial. In a preclinical study, non-human primates immunized with this Ad5 vector-based vaccine showed significant protection against the Ebola virus suggesting potential application for clinical studies in human subjects [36]. Further, Phase I clinical trials on healthy Chinese adults showed good safety and immunogenicity with the induction of specific antibodies and T-cell response within 28 days of one dose of vaccination. Based on the potential results from the phase I clinical studies, this vaccine was evaluated on a large population from Sierra Leone (severely affected by Ebola Virus in 2014) [36]. The authors observed a high humoral immune response of glycoprotein-specific antibodies in phase II clinical trials with a peak at the 28th day after injection of 8 × 10^10^ viral particles and of 1.6 × 10^11^ viral particles. Authors followed up this study up to 6 months and no difference in the antibody response was noted in volunteers after injection of 8 × 10^10^ and of 1.6 × 10^11^ viral particles [58]. Although the short durability of vaccine-elicited antibody indicated a prime booster regimen, the authors recommended the investigation of 8 × 10^10^ viral particles in phase III trials [58]. 

Due to the recent outbreak of COVID-19 caused by SARS-CoV-2, several studies have been undertaken worldwide to combat SARS-CoV-2. Zhu et al. studied safety, tolerability, and immunogenicity of Ad5 vectored COVID-19 vaccine expressing the spike glycoprotein of SARS-CoV-2 strain. The authors noted a significant increase in the neutralizing antibodies at day 14, and peak at day 28 after vaccination. They also observed peak response of specific T-cell at day 14 and suggested the requirement of further investigation of Ad vectored COVID-19 vaccine [58]. In other work, Folegatti et al. studied Chimpanzee-derived adenovirus-vectored vaccine (ChAdOx1 nCoV-19) expressing the SARS-CoV-2 spike protein in phase I/II, single-blind, randomized controlled trial. The authors reported an acceptable safety profile and increased antibody responses upon homologous boosting leading to induction of both humoral and cellular immune responses. Evaluation of this candidate vaccine in phase III clinical studies showed promising results [59], further leading to the development of ChAdOx1 nCoV-19 vaccine (Oxford/AstraZeneca), which is now authorized for human use [60]. In recent years, the chimpanzee-derived adenovirus vector has been widely investigated in vaccine research and development due to its unique feature of non-reactivity to pre-existing human adenovirus neutralizing antibodies [61]. Logunov et al. developed a heterologous COVID-19 vaccine consisting of two components, a recombinant Ad26 vector (rAd26) and a recombinant Ad5 vector (rAd5), both carrying the gene for SARS-CoV-2 spike glycoprotein (rAd26-S and rAd5-S) and evaluated in phase I/II clinical trials [62]. They also reported a good safety profile with the induction of strong humoral and cellular immune responses in participants. All these studies indicated that the adenovirus expresses a foreign gene as antigen/immunogen for the purpose of vaccine and thus it functions as both carrier and adjuvant. Several other viral vectors such as lentiviral [40,41,42], modified vaccinia virus Ankara (MVA) [43], cytomegalovirus (CMV) [23], poxvirus [22,24], measles virus [25,45], vesicular stomatitis virus [15,18], HSV and alphavirus [27] are also under different phases of clinical trials. Lentiviral and integrase defective lentiviral vector has been used widely in the vaccination strategies and several preclinical and phase I/II studies are currently undergoing [63,64,65]. In a Phase I/II clinical trial, Shenzhen Geno-Immune Medical Institute, China has proposed to develop a universal vaccine and test innovative COVID-19 minigenes engineered based on multiple viral genes, using an efficient lentiviral vector system (NHP/TYF) to express viral proteins and immune-modulatory genes to modify artificial APCs and to activate T cells [64]. 

MVA-vectored HIV-1 vaccine showed induction of modest vector-specific T cell responses in human subjects with excellent safety [66]. After two decades of preclinical trials, MVA vaccine vectors have recently been advanced into clinical trials with two Phase I studies completed HIV-1 [67], Chikungunya virus (CHIKV) [68]) whereas Phase II CHIKV trials are ongoing in Europe [69]. These preclinical and clinical studies indicate the usefulness of viral vectors in vaccination and a promise for future development. 

### 2.2. Virus-Like Particles (VLPs) and Virosomes

VLPs are empty, multiprotein, non-replicating and non-infectious structures resembling natural virions, which are prepared spontaneously during in vitro protein expression by the self-assembly mechanism of viral protein [70]. VLPs are considered a type of subunit vaccine due to the presence of a non-infectious subset of viral components. Another subunit vaccine (delivery system) similar to VLPs is called virosomes, which contains an envelope of mono or bilayer phospholipid vesicles to which additional components of the virus or pathogen/antigen, virus-derived proteins may be attached or inserted [71,72]. VLPs including virosomes exert their effect on APCs and on antigen-specific lymphocytes via their overall particle structure and individual components [73]. VLPs and virosomes have attracted researchers because they are structurally and morphologically similar to infectious viruses, retain the ability of infective viral particles to bind and penetrate the cell, and stimulate both humoral and cellular immunity. They are also safe and stable compared to killed/attenuated viral vaccines and soluble antigens [74]. Promising characteristics of VLPs and virosomes resulted in many marketed products (e.g., Hepatitis B virus (HBV), human papilloma virus, etc. and fruitful studies under different phases of clinical trials [70,75]. There are three generations of VLP vaccines against HBV. The first generation includes Heptavax-B, which is a hematogenous HBV vaccine made up of hepatitis B surface antigen VLP with a diameter of 22 nm. The second generations HBV vaccines are genetically engineered vaccines based on HBV VLP, namely Recombivax HB (The first licensed VLP against HBV) (developed by Merck) and (Engerix-B) (developed by Glaxo SmithKline) which composed of the viral small envelope protein from Saccharomyces cerevisiae system to stably express Hepatitis B surface antigen (HBsAg), producing particles of around 20 nm size. These vaccines are widely used today and considered more immunogenic than first-generation VLPs. Lastly, third-generation VLP includes Sci-B-Vac which contains three HBV surface antigens (S, pre-S1 and pre-S2 antigens) [75]. In a single-center prospective study recruiting 31 HIV-positive adults, the protection rate of Sci-B-Vac vaccine against HBV was found to be 84% after completion of three doses of vaccine without any serious side effects [76]. Subsequent studies demonstrated its safety and efficacy in over 500,000 patients. The Sci-B-Vac^®^ is currently approved in Israel and 14 other countries for commercial use [77], while US FDA has recently accepted its filing of the Biologics License Application (BLA). Another HBV vaccine called Heplisav-B (Hepatitis B Vaccine (recombinant) adjuvanted) is indicated for the prevention of infection caused by all known subtypes of hepatitis B virus and approved by the US FDA for use in adults. Heplisav-B is a 20 nm VLP self-assembly prepared from HBsAg and CpG sequence 1018 as an adjuvant [78]. The seroprotection rate of Heplisav-B was statistically significant that following Engerix-B at a 95% confidence interval [78]. It is supplied as a sterile solution for intramuscular injection. In addition, Merck received approval for a VLP vaccine, Gardasil^®^ (a quadrivalent HPV VLP vaccine) for immunization against human papillomavirus (HPV) and subsequent prevention of cervical cancer and genital warts [79]. Moreover, GlaxoSmithKline introduced Cervarix^®^ (a bivalent HPV VLP vaccine) vaccine for the prevention of cervical pre-cancer and cancer associated with oncogenic HPV types 16 and 18 [80]. Currently approved three prophylactic HPV vaccines in the market viz. Gardasil^®^, Gardasil-9^®^ (a nonvalent HPV VLP vaccine), Cervarix^®^ and Cecolin uses L1 major capsid protein, which self-assemble into VLPs to induce strong and specific anti-L1 VLP immune response, while next generation vaccines using L2 peptide are under investigation [81]. Primary mechanism of action of commercial VLP vaccines is to produce antibodies VLP parent virus specific antibodies, which further neutralize the parent virus to protect against infection.

The first virosome-based vaccine formulation was developed and patented by Berna Biologics Ltd. Switzerland and it is marketed as Epaxal^®^. Epaxal^®^ consists of formalin-inactivated Hepatitis A virus (HAV) (strain RG-SB), which has demonstrated tolerance and high immunogenicity with 88–97% seroprotection 2 weeks after a single injection. Unlike other vaccines, this vaccine formulation is devoid of aluminum hydroxide adjuvant suggesting the potential use of virosomes as a vaccine adjuvant delivery system [75,82]. Direct comparison of Epaxal with aluminum-adsorbed vaccine demonstrated similar immunogenicity with fewer local reactions [83]. Similarly, Inflexal^®^ V is another virosome-based adjuvanted influenza vaccine licensed for all age groups (up from 6 months) that was developed by Berna Biologics Ltd., Switzerland [9]. Inflexal^®^V is composed of a haemagglutinin surface molecule of the influenza virus, which is attached to the lecithin phospholipid bilayer virosome. The Inflexal^®^ V virosomes are spherical, unilamellar vesicles with a size of around 150 nm [82]. Mymetics, a US-registered biotechnology company has a pipeline of potential virosomal vaccines to malaria and influenza that are being tested in clinical trials [84]. Thus, advancements in the VLP and virosome technology have shown promising characteristics of these carrier systems to carry potential antigens to induce an effective immune response, which can be seen by already existing products in the market for clinical application.

### 2.3. Non-Viral Vectors

Non-viral vectors are often called delivery vehicles and typically consist of DNA (usually plasmid DNA produced in bacteria) or RNA as an antigen which is delivered to the target cell to elicit immune response [85]. In addition to nucleic acids, proteins and peptides antigens delivery by non-viral vectors also shows great potential in vaccine development. However, the potential drawbacks of endonuclease degradation, lower efficiency and repeated doses of naked DNA vaccine have been reduced using delivery vehicles. Therefore, natural or synthetic materials (lipids, polymers) have been utilized to prepare non-viral vectors which encapsulate or adsorbs antigen and fuse with the cell membrane to release it into the cytoplasm of the cell. Vaccination with non-viral vectors presents many advantages over viral vectors like safety and efficacy (due to absence of viral component), no limit to DNA insert size, ease in large scale production, low or no-host immunogenicity, protection of antigens, targeting, long-lasting gene expression and adjuvant effect [86]. However, certain disadvantages like low transfection efficiency, episomal expression, cellular toxicity, inflammation due to unmethylated CpG DNA sequences limit their use [26]. Some of the widely investigated adjuvants and delivery systems such as adjuvants, with appropriate preclinical and clinical examples, are discussed below. 

#### 2.3.1. Nanoemulsion-Based Adjuvants

##### MF59

MF59 is an oil-in-water (o/w) emulsion composed of squalene (4.3% *v*/*v*) (a naturally occurring substance) and two surfactants, polysorbate 80 (0.5% *v*/*v*, Tween 80) and sorbitan trioleate (0.5% *v*/*v*, Span 85) emulsified in citrate buffer leading to the formation of ~160 nm-sized droplets [87]. An MF59-adjuvanted seasonal influenza vaccine (Fluad^®^) was the first MF59 vaccine to be licensed, in 1997. Later, in 2009, an MF59-adjuvanted H1N1 pandemic influenza vaccines (Focetria^®^ and Celture^®^) were licensed and have been distributed to populations including pregnant women, young children and elderly people [88,89]. Though MF59 is a superior adjuvant than alum in inducing both antibody and T cell responses for the influenza vaccine, side-effects such as pain at the injection site and reactogenicity have been observed in some patients [90]. Despite these common side-effects of vaccines, there is continued interest by researchers in employing them as adjuvants. Recently, Yang et al. designed a vaccine consisting of OmpK/Omp22 fusion protein in combination with MF59 to protect against *Acinetobacter baumannii* infections. Intratracheal immunization along with two booster doses induced antigen-specific antibodies, lowered bacterial load in the blood and lung tissues, and controlled blood inflammatory cytokines resulting in a greater survival rate in BALB/c mice [91]. In another study, Chang et al. administered intramuscular single immunization of inactivated whole-virion H7N9 influenza vaccine in a mouse model. The vaccine successfully induced specific immunoglobulins (Ig), IgM and IgG titers as detected by ELISA suggesting effective protection against the H7N9 virus after a single dose [92]. These studies show the promise of MF59 as an effective adjuvant.

The chemical structure of muramyl dipeptide (MDP) can be modulated to enhance its adjuvant activity while limiting its pyrogenic side effects by using oil-in-water emulsion carriers such as MF59. Murabutide (MB), is a safe derivative of MDP, and is a squalene-based emulsion adjuvant that interacts with both innate and adaptive immune systems and induces its effect through activation of NOD2 (Nucleotide-binding oligomerization domain-containing protein 2) [93]. Kantipakala et al. used MB to formulate a vaccine delivery system with ovalbumin (OVA) as a model antigen. The MB-based system was rapidly uptaken by dendritic cells (DCs) and upon in vivo subcutaneous delivery triggered a 32-fold increase in OVA-specific IgG antibody titers and upregulated interleukins (IL) namely IL-2, IL-12 and interferons (IFN)-γ cytokines indicating Th1 (cells stimulate cellular immune response, participate in the inhibition of macrophage activation and stimulate B cells to produce IgM, IgG1 [94]) immune response in mice [95]. In another study, Feinen et al. tested the adjuvanticity of the murabutide and Advax, an adjuvant made from delta inulin, in murine pulmonary anthrax infection model based on recombinant protective antigen (PA). The combination induced a robust and enduring B-cell memory response post-aerosol challenge by *Bacillus anthracis* Sterne (strain 7702) as reflected by ~4-fold higher anti-PA IgG titers and 3-fold less inflammation than PA with Alhydrogel in female A/J mice [96]. 

##### Montanide™

SEPPIC Inc. (Paris, France) developed highly refined emulsifiers from the mannide monooleate family in a natural metabolizable oil solution, which were named as Montanide™ ISA 50V, 51, ISA 206, 720 [97]. Among these, ISA 50V, 51 and 720 are *w*/*o* emulsions while ISA 206 is a water-in-oil-in-water (*w*/*o*/*w*) double emulsion with particle size ranging between 10–500 nm. Although these adjuvants have been shown to induce a strong immune response, severe local reactions have limited their use. As per clinical studies, Montanides™ ISA 51 VG and 720 are safe for human use and induce CD4 and CD8 responses [98]. Savoji et al. formulated HBsAg in Montanide ISA 266 and compared the induction of cellular and humoral immune response with commercially available HBsAg/Alum HBV vaccine. In vivo immunization of BALB/c mice showed that the formulation of HBsAg/Montanide ISA-266 vaccine elevated IgG1 and IgG2a levels, as markers for Th2 (cells stimulate humoral immune response, promotes B cell proliferation and induces antibody production (IL-4) [99]) and Th1 patterns, than HBsAg/Alum. This confirmed the robust humoral response and protection from the protease enzyme by HBsAg/Montanide ISA 266 vaccine [100]. In another study, Tehrani et al. demonstrated the efficacy of thiol-specific antioxidant (TSA) antigen against *Leishmania major* using adjuvant Montanide ISA 70. In vivo immunization of BALB/c mice and challenge with *Leishmania major* revealed that specific IgG1 and IgG2a levels were elevated and vaccine-elicited humoral and cellular immune response [101]. Currently, Montanide™ adjuvanted vaccines, in particular ISA™51, against many diseases such as malaria, HIV, and various cancers are under different phases of clinical trials and have been reviewed in detail by van Doorn et al. [102].

#### 2.3.2. Lipid Nanocarriers

##### Immunostimulatory Complexes (ISCOMs)

ISCOMs were first described in 1984 by Morein et al. ISCOM adjuvants are particulate complexes containing protein antigen, saponin adjuvant (Quil A—which is derived from the bark of the South American *Quillaia saponaria* Molina tree), cholesterol, and phospholipids [28]. The cholesterol strongly interacts with saponin to form a unique cage-like particulate structure with a size of 40 nm, which is likely to contribute to the stability of the adjuvant and also reduces the hemolytic activity of the saponins which is important for its safety [9]. ISCOM complex traps the protein antigens (typically hydrophobic membrane proteins) through apolar interactions [103]. ISCOMs can bind and penetrate cellular membranes and helps deliver the immunogen into the cytosol of the target cell leading to endogenous processing and presentation of the immunogenic peptide via MHC-I for induction of CD8+ T cells [104]. Thus, ISCOMs represent good vehicles for intracellular delivery of DNA-based vaccines [105]. Pabreja et al. formulated a pulmonary tubercular vaccine using Antigen 85 complex (Ag85)-loaded systems such as the ISCOM. Immunological outcomes on BALB/c mice with ISCOMs containing Quil A showed a high level of IgG1 supporting significant development in humoral and cellular immune responses after pulmonary immunization [106]. Cibulski et al. investigated the immunological activity of ISCOMs formulated using a saponin derived from *Quillaja brasiliensis* (QB-90) termed IQB-90, consisting of cholesterol, phospholipid, and OVA as a model antigen. Subcutaneous administration of IQB-90 resulted in strong serum antibody response of specific IgG1 and IgG2 with effective T-cell proliferation and greater Th1 cytokines responses. Further, intranasal delivery evoked serum IgG, IgG1, and mucosal IgA responses in distal systemic sites providing an advantage over traditional ISCOMs based on Quil A [107]. The only concern while employing ISCOMs is the severe toxicity leading to hemolysis or granulomas [108].

##### Liposomes

Liposomes are spherical vesicles, with size ≤500 nm, composed of amphiphilic phospholipids and cholesterol, which self-associate into bilayers with an aqueous interior that can encapsulate many drug molecules including protein and DNA-based vaccines [33]. In the delivery of vaccines, the antigen (be it peptide, mRNA, or DNA) can either be adsorbed on the surface of the liposome, loaded in the liposome core, or the lipid bilayer. A large number of reports have demonstrated immunomodulatory effects such as depot effect and enhanced ingestion by APCs, of liposomes or suspensions of lipids and/or phospholipids when administered into the body as a vaccine adjuvant [109,110]. Passive targeting due to their particulate nature and tendency to interact with macrophages of the reticuloendothelial system is the likely mechanism of action by which liposomes exert their adjuvant effect [111]. Few liposome-based vaccine formulations are already approved (Epaxel, Inflexal, etc.) and several of them are in clinical trials [9,82]. The inclusion of a cationic compound in the liposomal formulation renders them positive and enhances the interaction with negatively charged cell membranes [112]. Varypataki et al. prepared cationic liposomes encapsulated with ovalbumin containing the model Cytotoxic T lymphocytes (CTLs) epitope SIINFEKL and TLR3 ligand. The subcutaneous and intradermal immunization of mice concluded that liposomal formulation was successful in inducing a functional CD8+T cell immune response with 25 fold increase over the free model CTL epitope and TLR3 ligand [113]. In addition, several studies have used a combination of dimethyl dioctadecyl ammonium (DDA) lipid-based liposomes to enhance immunity against influenza, chlamydia, erthrocytic-stage malaria and tuberculosis infections [109,114].

Liposomes enable efficient delivery of mRNA and have been extensively used to deliver both conventional and self-amplifying mRNA against infectious pathogens. Moyo et al. utilized a polyethyleneimine (PEI)-based self-amplifying mRNA vaccine encoding HIV-1 proteins to induce potent T cell responses in BALB/c mice. A single immunization induced polyfunctional CD4+ and CD8+ T cell responses that were maintained for at least 22 weeks post-immunization and controlled HIV-1 infection [115]. Dai et al. developed polyethylene imine (PEI) incorporated liposomes for the administration of the lipopeptide-based vaccine containing Group A *Streptococcus* (GAS J8) epitope (as B cell epitope) against GAS. Intranasal administration of mice by liposomal vaccine induced significant mucosal and systemic immunity by the production of IgA and IgG antibodies [116]. In another study, Huang et al. utilized Pfs230, a malaria transmission-blocking antigen, along with cobalt-porphyrin-phospholipid (CoPoP) liposomes for the development of a vaccine-adjuvant platform. In vivo immunization of CD-1 mice with Pfs230C1/CoPoP elicited IgG antibodies, induced higher IgG2-to-IgG1 and significantly reduced parasite transmission as compared to other treatment groups of Alum and Montanide ISA720. Further, intramuscular administration of New Zealand white female rabbits showed similar results with Pfs230C1/CoPoP treatment group eliciting antibodies and inhibiting parasite transmission [117]. The most recent application of liposomes as a drug delivery carrier and an adjuvant is Moderna’s candidate vaccine mRNA-1273 for COVID-19. It is a liposomal formulation encapsulated with nucleoside modified mRNA that encodes the SARS-CoV-2 spike (S) glycoprotein, a vital component of viral entry that allows viruses to penetrate host cells and cause infection [118]. The initial clinical trials demonstrated that the vaccine induced anti-SARS-CoV-2 immune response in all participants without significant toxicity [54]. The recently concluded Phase III clinical trial of Moderna’s vaccine candidate for COVID-19 showed 94.1% efficacy in preventing COVID-19 illness without any local or systemic toxicity, after a two-dose vaccination strategy [119]. Another example is a COVID-19 vaccine candidate by Pfizer and BioNTech, BNT162b2, comprising of lipid nanoparticle and encapsulated nucleoside modified RNA vaccine with encoded membrane-anchored SARS-CoV-2 spike protein. The two-dose vaccine regimen was shown to be 95% effective against COVID-19 infections with mild-to-moderate short-term side-effects making it available for mass vaccination [120]. In December 2020, Moderna’s and Pfizer-BioNTech’s COVID-19 vaccine received Emergency Use Authorization (EUA) by the US FDA to prevent coronavirus disease for use in individuals 18 years of age or older [121,122].

##### Biodegradable Polymeric Nanoparticles

Biodegradable polymeric NPs (PNPs) display interesting features related to the protection/stabilization of vaccine antigens until they reach the target site. A large number of polymers exists from which PNPs can be prepared, amongst which widely used polymers like (poly-(d,l-lactide-co-glycolide) (PLGA), poly(lactic acid) (PLA), Poly (alkyl cyanoacrylalte) (PACA), polyanhydrides and chitosan, etc., are already approved by the FDA for use in humans (e.g., as sutures, bone implants and screws as well as implants for sustained drug delivery) [123]. The biodegradable properties of these polymers make them promising vehicles for the exploration of antigen delivery; however, researchers are still striving for regulatory approval and these delivery systems have not yet entered into clinical trials for vaccine application as there are stability and dose optimization issues that need to be addressed. Depending upon their type, antigens can be either encapsulated or adsorbed on the surface of NPs. Encapsulation of antigen ensures its protection from the harmful gastro-intestinal environment, protects from drug degradation in the systemic circulation (by avoiding phagocytosis and releasing the drug in cytoplasm) and sustained release over a prolonged period of time, while adsorption of antigen on NPs avoids exposure to harmful organic solvents or acidic pH during the formulation process [124].

Various preclinical studies have indicated significant induction of antigen-specific immunity on the administration of PNPs. Polyanhydride-based nanoparticles and chitosan nanoparticles exhibit adjuvant-like properties by activating APCs and induce both humoral and cell-mediated immune responses on their own [37]. To investigate the influence of polyanhydride chemistry on immune response induction, Wafa et al. synthesized different compositions of polyanhydride copolymers consisting of 1,8-bis-(*p*-carboxyphenoxy)-3,6-dioxaoctane (CPTEG), 1,6-bis-(*p*-carboxyphenoxy)-hexane (CPH), and sebacic anhydride (SA). The biodegradable nanoparticles with three different compositions of polyanhydride (50:50 CPTEG:CPH, 20:80 CPTEG:CPH, and 20:80 CPH:SA) encapsulating OVA were administered subcutaneously in C57BL/6J mice. In vivo immunization with 20:80 CPTEG:CPH treatment group induced a high level of CD8+ T lymphocytes, serum titers of OVA-specific IgG antibodies and longer protection against tumor challenged with an OVA-expressing thymoma cell line than other treatment groups suggesting role of copolymer composition in stimulating immune response [38]. Liu et al. demonstrated antigen stability, antigenicity and release kinetics of MUC4β- 20:80 CPTEG:CPH nanovaccine. In vitro studies showed sustained release of MUC4β protein with minimal protein degradation and loss of epitope availability. In vivo immunization of nanovaccine induced MUC4β-specific IgG immune response by the synergistic effect of MUC4β and 20:80 CTPEG:CPH nanoparticles whereas unloaded nanoparticles failed to induce effective antibody response [39]. Using the previously mentioned copolymer composition of 20:80 CPTEG:CPH, Banerjee et al. formulated a pancreatic nanoparticle vaccine by encapsulating Mucin 4β (MUC4β), a glycoprotein overexpressed in pancreatic cancer. Nanovaccine demonstrated significant increase in the surface expression of MHC I and MHC II, costimulatory molecules (CD80, CD86), and the secretion of pro-inflammatory cytokines (IFN-γ, IL-6, and IL-12) in immature dendritic cells in vitro compared to MUC4β alone or MUC4β/blank nanoparticles. Further, in vivo immunization of C57BL/6 mice elicited higher IgG2b to IgG1 ratio along with a pro-inflammatory cytokine profile (IL-6, IL-12/ IL-23p40 and IFN-γ) suggesting a Th1-biased immune response [36]. Ross et al. demonstrated the immunogenicity of the recombinant H5 hemagglutinin trimer (H5_3_) encapsulated polyanhydride nanoparticles (PAN) against H5N1 influenza. Subcutaneous immunization consisting of prime and booster doses of nanovaccine enhanced CD4+T cell immune responses along with the induction of high neutralizing antibody titers in female BALB/c mice. Further, vaccinated mice were challenged with low-pathogenic H5N1 viral load and antibody response and body weight informatics show that the vaccine regimens induced protective immune responses without any significant difference in survival between naïve mice that were not challenged and challenged with virus mice treatment groups [125]. Similarly, Thukral et al. investigated mycobacterium antigens encapsulated PAN for treatment of Johne’s disease. The mycobacterium antigens are composed of culture filtrate (PAN-Cf) of *M. paratuberculosis* in nanovaccine. Female C57BL/6 mice were vaccinated subcutaneously and PAN-Cf vaccination group indicated induction of triple cytokines (IFN-γ, IL-2, TNF-α) producing CD8+ T cells in vivo. Following the viral challenge on mice post-vaccination, the continued production of cytokine secreting CD8+T cells in PAN-Cf vaccinated groups and a significant reduction in bacterial load, compared to animals that received inactivated vaccine, indicated the development of protective and sustained immunity against Johne’s disease [126].

Kunda et al. formulated a dry powder vaccine of PGA-co-PDL (poly(glycerol adipate-co-ω-pentadecalactone)) nanoformulation with particles size of ~150 nm containing antigen of *S. pneumoniae*, pneumococcal surface protein A (PspA), to be delivered via the pulmonary route. In the study, the authors immunized mice and found that mucosal immunization with formulation targeting the lungs was able to induce local and systemic antibodies, conferring protection against a *Streptococcus* strain expressing PspA from the homologous family 2 [127,128]. Li et al. engineered guanidyl-decorated PEG-PLA NPs (PECG) with ovalbumin for in vivo immunization. The authors demonstrated robust immune responses by regulating the secretion of cytokines including IFN-γ and tumor necrosis factor (TNF)-α via depot effect [129]. In another study by Gu and co-workers, utilized immunopotentiator Angelica sinensis polysaccharide (ASP) with PLGA nanoparticles surface functionalized with PEI and PCV2 antigen together as ASP-PLGA-PEI delivery system. In vivo immunization of female ICR mice enhanced the antigen uptake, activated macrophages and lead to the production of IL-1β and IL-12p70 cytokines. Further, ASP-PLGA-PEI with adsorbed PCV2 significantly induces PCV2-specific IgG immune response and cytokines level with mixed Th1/Th2 immune response suggesting an effective vaccine delivery [130]. Khademi et al. proposed a promising strategy for *Mycobacterium tuberculosis* vaccine consisting of HspX/EsxS-fused protein encapsulated in PLGA and DOTAP (1,2-dioleoyl-3-trimethylammonium propane). Subcutaneous immunization of BALB/c mice with the proposed vaccine showed higher levels FN-γ and IL-4 cytokines in splenocytes and serum anti-HspX/EsxS IgG1 and IgG2a titers as compared to conventional BCG (Bacillus Calmette–Guérin) vaccine [131].

The adjuvanticity of poly-ε-caprolactone/ chitosan nanoparticles containing HBsAg and plasmid DNA encoding HBsAg (pRC/CMV-HBs) was investigated by immunization of C57BL/6 mice. In vivo results showed that the vaccine induced strong anti-HBsAg IgG titers and antigen-specific IFN-γ and IL-17 secretion by spleen cells after subcutaneous vaccination [132]. In another study, anthrax vaccine was developed by Chaung and co-workers, consisting of fucoidan-quaternary chitosan nanoparticles and anthrax vaccine adsorbed (AVA) as adjuvants. In vivo immunization by this anthrax vaccine in A/J mice demonstrated higher IgG-anti-protective antigen titers compared to CpG ODN plus AVA or AVA alone. Further, to evaluate protection, the vaccinated mice were subjected to anthrax lethal toxin challenge and survival studies show a superior survival rate of chitosan plus AVA vaccination over the other two treatment groups [133]. In order to evaluate the efficacy of chitosan as adjuvants, El-Sissi et al. developed chitosan nanoparticles (CNP) using ionic gelation method and loaded them with Rift Valley Fever Virus (RVFV) inactivated antigen to fabricate RVFV-chitosan nanoparticles-based vaccine (RVFV-CNP). They also utilized other combinations of RVFV-based vaccines such as RVFV-chitosan (RVFV-CS), RVFV-Alum, and adjuvant-free RVFV. In vivo vaccination of Swiss albino mice validated the safety and stimulation of innate and adaptive immunity by upregulating IL-2, IFN-γ and IL-4 in RVFV-CS and RVFV-CNP treatment groups, with later demonstrating superior efficacy compared to other groups [29]. Biodegradable nanoparticles are advantageous as they can mimic the priming and boosting effect by modified release and potential for single-shot vaccines reducing the cost of vaccination.

New classes of materials such as dendrimers, cyclodextrins have also been explored in vaccine delivery. Dendrimers are highly biocompatible and exhibit predictable biodistribution and cell membrane interacting characteristics due to their size and surface charge. Their optimal characteristics make them efficient immunostimulating adjuvant that can increase the efficiency of vaccines [134]. Asgary et al. studied the adjuvanticity of G2 dendrimer. A nonlinear globular G2 dendrimer comprising citric acid and polyethylene glycol 600 (PEG-600) was synthesized and the adjuvanticity was evaluated in a mice model after administration with the rabies virus inactivated vaccine. They showed that dendrimer-based formulations enhanced immune responses, high neutralizing antibodies against rabies virus, and higher survival rate of mice [135]. Chahal and Khan et al. synthesized dendrimer molecule encapsulating antigen-expressing replicon mRNAs for adjuvant-free nanoparticle vaccine. In vivo intramuscular immunization elicited CD8+T cells and antibody responses against various lethal pathogens such as the Ebola virus, H1N1 virus influenza and *Toxoplasma gondii* [136].

##### Non-Biodegradable NPs

Various non-biodegradable materials such as gold [137], carbon [138], silica [139], quantum dots [140] and polystyrene [141] have been utilized as vaccine delivery systems and adjuvants. They remain in the tissue for an extended period of time and can thus present the antigen to tissues with enhanced immunogenicity [9]. In general, this is achieved by surface functionalization of nanomaterial to target specific cells. Although non-biodegradable materials facilitate conjugation with different functional groups and antigens, induce higher cellular and humoral response but often lead to toxicity and aggregation in tissues requiring further validation of safety [142]. Gold nanoparticles (GNPs) are efficient adjuvant and delivery vehicles with or without surface functionalization. GNPs have been explored for in vivo delivery of plasmid DNA for HIV treatment. Xu et al. formulated gold nanorods modified with poly(diallydimethylammoniumchloride) or polyethyleneimine that significantly promoted cellular and humoral immunity as well as T-cell proliferation, through activating antigen-presenting cells, when compared to naked HIV envelope plasmid DNA treatment in vivo [143]. Wang et al. conjugated recombinant trimetric influenza hemagglutinin on gold nanoparticles, coupled with TLR-5 agonist flagellin as a particulate adjuvant system. Intranasal vaccination in mice increased influenza-specific IgA and IgG levels, and led to antigen-specific IFN-γ secreting CD4+ cell proliferation and activated CD8+T cells [35].

Oxidized carbon nanosphere (OCN), a negatively charged carbon nanoparticle was formulated and investigated for in vitro antigen uptake and ability to generate in vivo immune response. Subcutaneous immunization of BALB/c mice with OCN and OVA as a model antigen, demonstrated improved cell-mediated immune response by elevating antigen-specific CD8+ T cells while localizing in MHC class I compartments [144]. The potential of quantum dots (QDs) as fluorescent nanoparticles for in vitro and in vivo imaging of DCs is widely explored whereas an antigen-delivery system to enhance DC-mediated immune responses is being investigated. In a recent study, a group of researchers developed graphene QDs to access immune and central nervous cells during neuroinflammation and generate an encephalitogenic Th1 immune response. Intraperitoneal administration resulted in the inhibition of INF-γ-expressing T-cells and attenuated autoimmune encephalomyelitis by modulating mitogen-activated protein kinases (MAPK)/Atk signaling in rats [145].

##### Calcium Phosphate NPs (CPNPs)

Calcium phosphate has been used for over 30 years to deliver genetic material to mammalian cells. It has good biocompatibility as it is a naturally occurring, readily absorbed normal body constituent and thus overcomes safety-related issues [146]. It was used until the 1980s as an adjuvant in childhood diphtheria–tetanus–pertussis (DTP) vaccine formulations in France, in the form of calcium phosphate gel or suspension [147]. Several preclinical studies have shown that functionalized calcium phosphate nanoparticles (100–400 nm) are capable of inducing both innate and adaptive immunity by activation of dendritic cells [148]. BioSante Pharmaceuticals, Inc. demonstrated a better immunostimulatory effect of CPNPs in comparison with the commonly used aluminum (alum) adjuvants for HSV-2 and Epstein–Barr virus (EBV) infections [149]. CPNPs are investigated as an alternative to aluminum as an adjuvant for many vaccines. In vivo experiments showed that micrometer-sized CPNP aggregates generated high titer of neutralizing antibody in BALB/c mice, and showed high protection against HSV-type 2 infection, which is more potent than aluminum adjuvant [125]. Murcol et al. used CPNP vaccine adjuvant to formulate an inactivated whole virus influenza H1 N1 vaccine as a potential dose-sparing strategy. In vivo studies in BALB/c mice showed significantly higher hemagglutination inhibition, virus neutralization and IgG antibody titers than non-adjuvanted vaccines [150]. Kopp et al. fabricated CPNPs with surface conjugated synthetic peptide P1 and P2 against HSV-1 infections. The P1 and P2 peptides mimic a neutralizing epitope on the HSV-1/2 gB, known to elicit a protective antibody response in mice. The booster immunization of this vaccine led to the production of cell-to-cell spread inhibiting antibodies and protection from genital infection and blindness in animal models [151].

##### Colloidally Stable Nanoparticles

Variety of carbohydrates such as dextran, pullulan and mannose, etc., cannot self-associate in an aqueous solution due to their water solubility. They can be conjugated to hydrophobic materials (e.g., cholesterol) to make them amphiphilic. Self-assembling of these molecules (with and without proteins) leads to the formation of colloidally stable nanoparticles with a size of 30–40 nm. The size, density and colloidal stability of the nanoparticles can be controlled by changing the substitution degree of hydrophobes and the hydrophobicity. Pullulan is a widely used water-soluble, neutral linear polysaccharide consisting of α–1, 6-linked maltotriose residues. Film-forming properties of Pullulan helps to entrap biological molecules and its excellent oxygen barrier properties make it stable with enhanced shelf-life. The mechanism of innate immunity depends on the binding of polysaccharides to mannose-binding lectins and other C-type lectins of the mannose receptor family on macrophages and DCs [152]. Various cholesterol-bearing pullulans with different molecular weights of the parent pullulan and degrees of substitution of the cholesteryl moiety have been described in the literature [153]. Recently, Nagatomo et al. prepared cholesteryl pullulan nanoparticles by encapsulating TNF-α for nasal delivery of the H1N1 influenza vaccine. In vivo immunization induced systemic IgG1 and mucosal IgA responses against a lethal challenge of A/PR/8/34 (H1N1) influenza virus. The mechanistic studies suggested elevated antigen uptake by DCs and activation of both B and T cells by expression of inflammation-related genes in nasopharynx lymphoid tissues [154].

##### Proteosomes

The proteosome consists of hydrophobic, proteinaceous nanoparticles (~150 nm), composed of major outer membrane proteins (OMPs) of *Neisseria meningitidis* [155]. OMPs have been successfully used in a marketed meningococcal vaccine (Menomune ^®^, Sanofi Pasteur) since 1981. Hydrophobic OMP is a good system for the delivery of apolar or amphiphilic antigens due to noncovalent interaction between the proteosome and antigen leading to the formation of appropriate complexes [9]. Various human clinical trials have qualified proteosomes as safe and well-tolerated materials for human use, mainly after intranasal administration [156]. FluINsure is an intranasal influenza vaccine composed of inactivated antigens with proteosome adjuvant and showed a good safety profile and induces both humoral and cellular immune responses in adults [157]. Menactra^®^ (Sanofi) is a quadrivalent conjugate vaccine containing four meningococcal polysaccharides conjugated to diphtheria toxoid that has also been marketed [158]. Phase I and II clinical studies results of proteosomes-adjuvanted trivalent inactivated influenza vaccine was safe and tolerable inducing systemic and mucosal immune responses after intranasal administration [159].

#### 2.3.3. Adjuvants Targeting Pattern Recognition Receptors (PRRs)

The immune system is equipped with receptors known as pattern recognition receptors (PRRs) which are specialized in their recognition to detect pathogens. These receptors are a vital element of the innate immune system and are mainly expressed by APCs such as DCs and macrophages, but they are also found in other immune and non-immune cells. Pathogens such as bacteria, viruses, fungi, and parasites express conserved sets of molecular patterns referred to as pathogen-associated molecular patterns (PAMPs). APCs recognize PAMPs through membrane-bound and intracellular receptors known as PRRs. Interaction between PAMPs and PRRs initiates a cascade of intracellular signaling pathways that promotes the production and secretion of pro-inflammatory cytokines such as type I IFN, TNF-α, IL-1 and IL-6 and chemokines including IL-8 and RANTES [160]. The inflammatory responses lead to the migration of mature DCs to lymph nodes and initiate adaptive immune responses leading to an immunological memory [161,162]. Adjuvants interact with cellular PRRs and stimulate innate immunity. Presently, numerous PRRs have been identified, including the TLRs, retinoic acid-inducible gene I (RIG-I)-like receptors (RLRs), nucleotide-binding oligomerization domain NOD-like receptors (NLRs), C-type lectin receptors (CLRs) and cytosolic DNA sensors (CDSs). Below, we described widely investigated PRRs and discuss examples wherein adjuvants targeted PRRs to generate a robust immune response.

##### C-Type Lectin Receptors (CLRs)

CLRs are transmembrane receptors that recognize carbohydrate structures represented by various pathogens. CLR family covers different types of receptors such as DC-SIGN, DEC-205, Dectin-1 and 2, and Mincle. They are capable of independently inducing immunity by lysosomal degradation and antigen presentation or deliver powerful signals via crosstalk to modulate responses triggered by other PRRs [163].

Nanocarriers stimulate CLRs in DCs and can significantly promote internalization and antigen presentation. For this purpose, mannose-appended pH-responsive nanoliposomes carrying OVA antigen were formulated to interact with APCs. In vivo immunization of C57BL/6 mice showed a significant increase in the IL-2, IL-10, IL-12 levels and serum IgG antibody responses. Mannose anchored liposomes specifically bind to the CLR receptor on APCs, promote internalization and present antigens (OVA) on MHC class I and II molecules [164]. Haro and Dyevoich et al. demonstrated that intraperitoneal injection of TLR and CLR agonist pairing of monophosphoryl lipid A (MPLA) and trehalose-6,6′-dicorynomycolate (TDCM) significantly inhibited tumor growth, ascites development and mortality associated with peritoneal carcinomatosis and lymphomatosis in mouse models [165]. Similarly, Phipps et al. formulated TLR and CLR agonist pairing adjuvant and co-administered with Pneumovax vaccine. The vaccine combination significantly elevated T-cell independent type-2 antigens and also improved protective efficacy in pneumococcal respiratory infection mouse models [166]. Recently, researchers examined the crosstalk of miR-511-3p, encoded within the human MRC1 gene, and CLRs on DCs. The downregulation of miR-511-3p results in triggering IL-10, IL-4 secretion while suppressing IL-17 with autologous T cells. Further, T-cells polarization and immune response modulation were controlled through regulating mannose receptors and DC-SIGN expression of the CLRs [167].

##### Toll-Like Receptor (TLRs)

Toll-like receptors (TLRs) are integral membrane-bound receptors that are essential for innate immunity and assist to shape the adaptive immune response. TLRs are triggered by various PAMPs (includes LPS) and danger-assisted molecular patterns (DAMPs). PAMPs or DAMPs can engage a variety of TLRs based on their position in the cell and/or made available after pathogen endocytosis or replication. Owing to their versatile functions, TLRs are considered the first line of immune defense [168,169,170]. The number of functional TLRs can differ in mammals, however, they all have conserved functions of activating inflammatory mediators. Humans have 10 TLRs; TLR2 (heterodimerizes with TLR1 or TLR6), and TLR4/5/10 are present at the cell membrane whereas TLR3/7/8/9 are functionally localized to endosomes [171]. TLRs contain three domains; an extracellular domain for sensing the ligand, transmembrane domain to anchor the TLR within membranes, and Toll/interleukin-1 receptor (TIR) domain to interact with other TIR-containing adaptors to initiate signaling [172,173].

TLR4 agonist (Monophosphoryl lipid A): Monophosphoryl lipid A (MPLA) is an agonist of TLR4 receptor developed by GlaxoSmithKline (GSK) for use as a vaccine adjuvant [174]. It is composed of detoxified lipopolysaccharide (LPS) from *Salmonella minnesota* R595. Many studies indicated that the LPS—a major component of the cell wall of Gram-negative bacteria—is responsible for both adjuvant and toxic effects [175]. The removal of the phosphate group from the reducing end sugar of the lipid A disaccharide led to 100 to 1000-fold decreased toxicity of the molecule while still retaining the immunostimulating activity. The resulting derivative having only one phosphate group was named monophosphoryl lipid A (MPL^®^) [174]. Other novel adjuvants and delivery vehicles containing MPL^®^ (AS04 and AS02A) have also been developed in combination with either aluminum salts or QS-21 (a purified component of the Quil A) [9]. These combinations have been used for HSV, HBV, *Streptococcus pneumoniae*, malaria and human papilloma virus (HPV). AS04 is recently licensed in Europe as a component of an improved vaccine for hepatitis B (Fendrix) [176]. Tian et al. developed a DNA vaccine against *Mycobacterium tuberculosis* using DMT liposomal adjuvant system consisting of dimethyldioctadecylammonium (DDA), MPLA and trehalose 6,6′-dibehenate (TDB). Further, to improve the efficacy of the DMT vaccine, the authors prepared a complex of DMT and plasmid pCMFO which secretes the combination of four multistage antigens (Rv2875, Rv3044, Rv2073c, and Rv0577) of *M. tuberculosis*. Immunization of female C57BL/6 followed by *M. tuberculosis* aerosol infection studies confirmed that the formulated vaccine provides enhanced and persistent protection by eliciting CFMO-specific IL-2+ T cell responses in vivo [177].

TLR7 and TLR8 agonist: Dowling et al. conjugated Imidazoquinoline, a TLR-8 agonist, to poly(ethylene glycol)-bl-poly(propylene sulfide) (PEG-bl-PPS) nanocarrier which increased activation and maturation of naïve dendritic cells due to selective endocytosis and prolonged immunogen by the nanocarrier [178]. Buonsanti et al. introduced a new type of vaccine based on TLR7 agonist adsorbed to alum (Alum-TLR7) to potentiate the immune response to glycoconjugate vaccines in humans. They investigated Alum-TLR7 in a mouse model and results show improved potency of a CRM_197_-MenC by increasing anti-MenC antibody titers and serum bactericidal activity against MenC after a single immunization with a low dose of antigen [179].

TLR9 agonists (CpG oligodeoxynucleotides): Cytosine-phosphorothioate-guanine oligodeoxynucleotides (CpG ODN) have sequence patterns like those found in bacterial DNA, activate potent cell-mediated immune responses, and hence are used for the treatment of various diseases [180]. CpG ODN is usually used either as a standalone molecule or as an adjuvant to alternative therapies. The unmethylated CpG dinucleotide flanked by two 5′ purines and two 3′ pyrimidines, present within the bacterial DNA is responsible for triggering immune responses [181]. CpG ODN are taken up by cells via adsorptive endocytosis and bind to the TLR9 present within the endosomes of B cells and plasmacytoid dendritic cells [182]. The binding then triggers a cascade of events such as maturation, differentiation and proliferation of multiple immune cells (B and T lymphocytes, macrophages, natural killer cells and monocytes/macrophages that produce IL-1, 6, 12, 18, IFN-γ and TNF-α) [34,180].

CpG ODN can be conjugated with protein or peptides for nanomedicines and is broadly divided into two types, covalently bonded or through electrostatic interactions between CpG ODN and protein/peptide. In the first type, covalently bonding CpG ODN to an antigen or allergen enables uptake of the adjuvant CpG ODN into the same antigen-presenting cell that has taken up the antigen. The typical procedure for covalently bonding a CpG ODN to a protein /peptide is to modify CpG ODN with a thiol group and protein/peptide with maleimide group and utilize the cross-linking of these groups. Alternatively, CpG ODN nanomedicines are also used and they are formulated by either encapsulating CpG ODN in nanomaterials or CpG ODN is surface-bound to nanomaterials. Goldinger et al. administered MelQbG10, VLP nanocarriers loaded with A-type CpG-oligonucleotide (CpG-ODN) and in combination with Melan-A/MART-1. In phase IIa clinical trials, stage III-IV melanoma patients were treated with MelQbG10 and topical Imiquimod and found to induce strong memory and effector CD8+ T-cell response. Here, CpG-ODN G10 facilitates T-cell activation and further triggers B-cells via TLR-9 whereas, Imiquimod activates APCs via TLR-7 [183]. Naito et al. developed the nasal vaccine consisting of recombinant PcrV adjuvanted CpG ODN against *P. aeruginosa* pneumonia and compared it with PcrV/Alum vaccine. Briefly, Institute of Cancer Research (ICR) mice were classified into treatment groups of PcrV-CpG, PcrV-Alum, PcrV, CpG and Alum and in vivo studies of IgA and IgG isotope titers supported the superiority of PcrV-CpG vaccinated. The intratracheal infection with a lethal dose of *P. aeruginosa* to challenge the vaccinated mice demonstrated a greater survival rate and disease protection by upregulation of PcrV-specific IgA titers in mice [184]. Tateishi et al. investigated the protective efficacy of the A/California/7/2009 (Cal7) spilt vaccine (X179A) along with CpG ODN (G9.1) after nasal and subcutaneous administration. After in vivo immunization with prime and booster dose of the vaccine, mice were challenged with Cal7 influenza virus where nasally delivered vaccine showed better protection and higher recovery rate from an infection as compared to subcutaneous vaccination. These results show decreased levels of type I IFN-associated protein and transcription factor-specific mRNA expression suggesting that vaccine elicits influenza virus-specific mucosal secretory IgA and serum IgG antibody responses without inducing inflammatory responses [185]. Nikitezuk et al. studied the preventive and therapeutic effects on E.G7-OVA tumors of PLGA particles by encapsulating both OVA and CpG-B ODN. The mice pre-administered with complex PLGA particles showed inhibition in tumor growth and a higher survival rate than in nontreated mice. In these mice, the OVA-specific Th1 responses were activated by the complexed PLGA particles, but there was no increased activation of OVA-specific CTLs, indicating that tumor growth inhibition could be due to Th1 responses [186]. Zhao et al. demonstrated that carbon nanotubes (CNT) enhance CpG ODN uptake and potentiate antiglioma immunity in mice. The CNT-CpG-B complex was formed by surface bonding and when administered into mice, it showed greater inhibition of tumor growth and a higher survival rate compared with the free CpG-B ODN administration. This implies that the complexation with CNTs improves the efficiency of CpG-B ODN uptake and causes activation of CTLs and natural killer cells [187]. Table 2 below lists various approved and clinically tested vaccines that use nanoparticle based adjuvants and delivery systems in their formulations.

## 3. Summary

The development of vaccines is one of the most successful interventions that have helped to reduce the burden of many diseases worldwide. Along with the advancement in vaccine development, adjuvants have also been of great interest due to their ability to induce stronger and more efficacious immune responses as compared to the immune response achieved using vaccines alone. An advanced understanding of clinical immunology in the past few decades has greatly influenced the interpretation of the mechanism of actions of adjuvants and ways to improve their efficacy. The development of vaccines and adjuvants is further influenced by the introduction of nanotechnology, which contributed in developing efficient vaccine adjuvants and delivery systems. In view of this, the present review discusses various adjuvants such as viral vectors, virus-like particles and virosomes; non-viral vectors namely nanoemulsions, lipid nanocarriers, biodegradable and non-degradable nanoparticles, calcium phosphate nanoparticles, colloidally stable nanoparticles, proteosomes; and pattern recognition receptors (PRR) covering c-type lectin receptors (CLR) and toll-like receptors (TLR) in the area of vaccine development. It is observed that adjuvants currently used in humans enhance humoral immunity, but many new adjuvants such as MVA, cationic liposomes, ISCOMs, and biodegradable and non-biodegradable nanoparticle vectored vaccine have shown to enhance specific T cell responses in pre-clinical and/or clinical development with excellent safety. The discovery of immunostimulatory agents like PRRs is also gaining great importance due to their ability to generate a precise tailor-made protective immune response. Thus, vaccine developers can now choose suitable adjuvants to achieve beneficial effects of vaccine adjuvants, e.g., (i) MF59 for influenza vaccines to attain effectiveness of vaccine at a smaller dose of antigen, (ii) AS04 for hepatitis B vaccine to achieve effectiveness by increasing the speed and reducing the number of immunizations required, and (iii) MF59 for influenza vaccines to broaden the range of antibody responses. This shows the diversity of mechanisms of the well-studied adjuvants may contribute to dealing with challenging diseases such as HIV, malaria, etc. Since the availability of a plethora of adjuvants, scientists engaged in the development of vaccines can now choose adjuvants with different properties as per the requirement. One such recent breakthrough is the approval of a vaccine to combat SARS-CoV-2 in a short duration of time. Thus, considering the overall development in the vaccines and adjuvants, eradication of many infectious of global concern will not be too optimistic to think about.

## 4. Future Challenges and Perspectives

The application of nanotechnology in vaccinology is rising as the pillar of the health care system. The enormous growth in this field is changing the world and the way we live by creating new scientific applications that are smaller, faster, safer, and more reliable, encompassing new medical treatments by creating new vaccines, adjuvants and vaccine-delivery techniques. The encouraging results obtained while exploring nano-sized vaccines, adjuvants and delivery systems have caught the imagination of researchers and vaccine manufacturers alike. As a result, there has been an explosion of research reports and patents on adjuvants and delivery systems as adjuvants for vaccine delivery. Further, few nanoscale-based vaccine delivery products have already made their way to market which includes viral vectored, virus-like particle, virosomes and MF59 adjuvanted vaccines. Vaccine delivery systems such as liposomes are also popular among researchers due to their several advantages some of which include the ability to attach targeting moieties to direct them to APCs, precise control over particle size, the inclusion of multiple antigens and adjuvants, and the ability to provide a depot effect. The most recent adjuvants, specifically those that target PRRs, can be used to tailor the immune response toward Th1/Th2. However, most of the advances still remain experimental (e.g., polymeric nanoparticles), and the regulatory agency approval issues are still more critical as it involves the complexities of proving the safety and efficacy of the vaccine, adjuvants and the vaccine delivery systems owing to nano-sized particles. In view of this, the FDA, and the European Medicines Evaluation Agency (EMEA) have taken the initiative to identify possible scientific and regulatory challenges. Therefore, if these issues are sorted out, many nanotechnology-based adjuvants and vaccine delivery systems are expected to serve society. In conclusion, researchers working in the field of vaccinology have plenty of choices for adjuvants with a variety of properties based on the type of protective immune response one wants to obtain. Further advancements will certainly open new avenues in the field of immunization.

## Figures and Tables

**Table 1:** Mode of action of adjuvants and delivery vehicles.

Type of Mechanism	Representative Materials *		Proposed Mechanism	References
	Antigen Examples	Adjuvant Examples		
Depot formation at the site of injection	Diphtheria toxoid, Hepatitis A, B	Alum, w/o emulsion, MPLA, biodegradable particles	Slow release, enhanced antigen uptake and presentation by APCs	[19,20,21,22,23,24,25,26,27,28,29]
Recruitment of innate immune cell	Diphtheria toxoid, Hepatitis A, HBsAg, HPV	Alum, MF59, w/o emulsion, CpG-ODN, particulate adjuvants	Upregulation of CTK and CMK, cellular recruitment at the site of injection	[19,20,22,23,24,25,26,27,28,30,31]
Antigen presentation/targeting	Diphtheria toxoid, Hepatitis A, HBsAg, HPV	Alum, MF59, w/o emulsion, CpG-ODN, particulate adjuvants (polymer-PLGA)	Targeting antigen to APC, uptake of antigen through PRRs on surface (TLRs and CLRs) and intracellularly (NLRs and RLRs), Dendritic cell activation, MHC class II expression	[19,20,22,24,25,26,27,29,30]
Activation of inflammasomes	Diphtheria toxoid	Alum, LPS, particulate adjuvants, DAP, MDP, MF59	Activation of PRRs-NLRs (NODs) and MHC II transactivator	[19,20,22,23,24,25,26,27,28,32,33]
Activation and maturation of APCs	Hepatitis A vaccines, influenza vaccine	LPS, liposomes, DOTAP, CpG-ODN, MF59, AS04, α-GAL, TDM, TDB	Maturation of DC’s-upregulation of CD40, CD54, CD80, CD83, CD86 and MHC class II molecules	[19,20,21,23,24,25,26,27,34,35]
Immunomodulation/CTL induction	Hepatitis A vaccines, influenza vaccine	LPS, liposomes, DOTAP, CpG-ODN, MF59, AS04, α-GAL, TDM, TDB	enhanced ability of APCs to induce T lymphocyte activation and differentiation, B cell (Humoral) and CD8+ cell responses (adaptive) immunity	[19,20,23,24,25,26,27,36,37,38,39]

Table Abbreviations: water-in-oil (w/o), Monophosphory lipid A(MPL A); Antigen presenting cell (APC); Dendritic cell (DC); Cytotoxic T-lymphocyte (CTL); Major Histocompatibility complex (MHC); Pathogen-recognition receptors (PRR); C-type lectin-like receptors (CLRs); Nucleotide oligomerization domain (NOD) like receptors (NLRs); Toll-like receptors (TLR); RLR (RIG-1 like receptors); lipopolysaccharides (LPS); α-galactosylceramide (α-GAL); Trehalose-6-6-dimycolate (TDM); Trehalose-6-6-dibehenate (TDB); Poly-lactic-co-glycolic acid (PLGA), Human serum albumin (HSA); Diaminopimelic acid (DAP); Muramyl Dipeptide (MDP); Cytokine (CTK); Chemokine (CMK), 1,2-dioleoyl-3-trimethylammonium propane (DOTAP), cytosine phosphoguanine deoxynucleoties (CpG DNA). (* representative list of antigens and adjuvants but are not limited to this).

**Table 2:** Approved and clinically tested vaccines using nanocarrier-based adjuvants and delivery systems.

Product	Application	Adjuvants Used	Approval Year, Company, Status of Research	Ref
**Viral Vectored Vaccines**				
ACAM2000	Smallpox	MVA-BN	2007, Sanofi Pasteur Biologics Co., Cambridge, MA, USA	[31]
Chimpanzee adenovirus vector (ChAdOx1)	Severe acute respiratory syndrome coronavirus 2 (SARS-CoV-2), Coronavirus disease (COVID-19)	Chimpanzee Adenoviral vector	2020, University of Oxford in collaboration with AstraZeneca, Cambridge, UK	[59]
Sputnik V (Gam-Covid-Vac)	SARS-CoV-2, COVID-19	Replication-deficient Ad types 5 and 26 vectors	2020, Gamaleya Research Insitute, Acellena Contract Drug Research and Development, Moscow, Russia	[188]
COVISHIELD™ (ChAdOx1)	SARS-CoV-2, COVID-19	Chimpanzee Adenoviral vector	2020, Serum Institute of India Pvt. Ltd., Pune, Maharashtra, India	[189]
Convidicea (Ad5nCoV)	SARS-CoV-2, COVID-19	Recombinant Adenoviral vector, Ad5	2020, CanSino Biologics, Tianjin China (approved for use in Mexico, China)	[190]
Janssen COVID‑19 Vaccine (Ad26)	SARS-CoV-2, COVID-19	Adenoviral vector, Ad 26	2021, Janssen Biotech, Inc., Horsham, PA, USA (Emergency use authorization by US FDA)	[191]
**Virus Like Particles**				
Recombivax HB^®^	Hepatitis B Virus (HBV)	Amorphous aluminum hydroxyphosphate sulfate	1986, Merck and Co. Inc., Kenilworth, NJ, USA	[75]
Engerix-B	HBV	Aluminum hydroxide	1989, Glaxo Smithkline (GSK), Middlesex, UK	[75]
Gardasil^®^	Human papillomavirus (HPV), cervical cancer and genital warts	Hydroxyphosphate sulphate	2006, Merck and Co. Inc., Kenilworth, NJ, USA	[75]
Cervarix	HPV	AS04 (aluminum hydroxide and MPLA)	2009, Glaxo Smithkline Biologicals SA, Rixensart, Belgium	[75]
Hecolin	Hepatitis E Virus (HEV)	Aluminum hydroxide	2011, Xiamen Innovax Biotech, Xiamen, Fujian, China	[75]
Gardasil-9^®^	HPV	Hydroxyphosphate sulphate	2014, Merck and Co. Inc., Kenilworth, NJ, USA	[75]
Heplisav-B	HBV	1018 ISS CpG ODN	2017, Dynavax Technologies Corporation, Emeryville, CA, USA	[75]
Sci-B-Vac^®^	HBV	Aluminum hydroxide	2020 (under regulatory approval process) VBI Vaccines Inc., Cambridge, MA, USA	[75]
Mosquirixs	Malaria and HBV	AS01 (MPL and Quillaja saponaria 21 (QS21))	2015, GlaxoSmithKline Biologicals S.A., Rixensart, Belgium	[75]
**Virosome-based vaccine**				
Epaxal™	Hepatitis A virus (HAV)	IRIV	1994, Berna Biotech Ltd., Berne, Switzerland	[73]
Inflexal^®^V	Influenza vaccine	IRIV	1997, Berna Biotech Ltd., Berne, Switzerland	[73]
Invivac^®^	Influenza vaccine	IRIV	2004, Solvay Pharmaceuticals B.V., DA Weesp, The Netherlands	[73]
NasalFlu^®^	Influenza vaccine	IRIV	2001, Berna Biotech Ltd., Berne, Switzerland	[73]
Epaxal Junior™	Novel pandemic A influenza virus (H1N1)	IRIV	1994, Berna Biotech Ltd., Berne, Switzerland.	[73]
**Non-viral vectored Vaccines**				
Celtura^®^	H1N1	MF59	2009, Novartis AG, Basel, Switzerland	[32]
Fluad^®^	Seasonal influenza in infants and young children	MF59	1997, Novartis AG, Basel, Switzerland Phase III Trials Completed 2010-11	[21,192]
Aflunov^®^	Pre-pandemic influenza (H5N1)	MF59	2010, Seqirus S.R.L., Monteriggioni, SI, Italy	[193]
Montanide	Malaria, HIV, cancer	MF59	Under clinical trial	[194]
FENDRIX	HBV	Aluminum phosphate and MPLA	2005, GlaxoSmithKline Biologicals., Rixensart, Belgium	[176,195]
Stimuvax^®^	Lung, breast, prostate and colorectal cancer	Liposome, MPLA	Merck KGaA, Darmstadt, Germany, Phase III Clinical Trial Completed	[196]
mRNA-1273	COVID-19	Liposome	2020, Moderna, Cambridge, MA, USA	[54]
BNT162b2	COVID-19	Liposome	2020, Pfizer, New York, NY, USA and BioNTech, Mainz, Rhineland-Palatinate, Germany	[122]
Prevnar^®^	Invasive Pneumococcal disease	Aluminum phosphate	2000, Wyeth Pharmaceuuticals, Madison, NJ, USA	[197]
Menactra^®^	Meningococcal disease	Aluminum	2005, Sanofi Pasteur, Lyon, France	[158]

Table Abbreviations: Severe acute respiratory syndrome coronavirus 2 (SARS-CoV-2), Coronavirus disease (COVID-19), Modified Vaccinia Ankara-BN (MVA-BN), Chimpanzee adenovirus vector (ChAdOx1), Adenoviral vector (Ad 26), Hepatitis B Virus (HBV), Human papillomavirus (HPV), Hepatitis E Virus (HEV) Immunopotentiating reconstituted influenza virosome (IRIV), Novel pandemic A influenza virus (H1N1), Pre-pandemic influenza (H5N1), Monophosphoryl lipid A (MPLA), Cytosine-phosphorothioate-guanine oligodeoxynucleotides (CpG ODN), Immunopotentiating reconstituted influenza virosome (IRIV), Modified Vaccinia Ankara-Bavarian Nordic (MVA-BN).
